# Supplementary material for: Utilizing evolutionary conservation to detect deleterious mutations and improve genomic prediction in cassava
Source: Front Plant Sci. 2023 Jan 9;13:1041925. doi: 10.3389/fpls.2022.1041925 (PMC10112518; doi:10.3389/fpls.2022.1041925)
Supplement: Supplementary file 1 [file Image_1.pdf]

Supplemental Figures

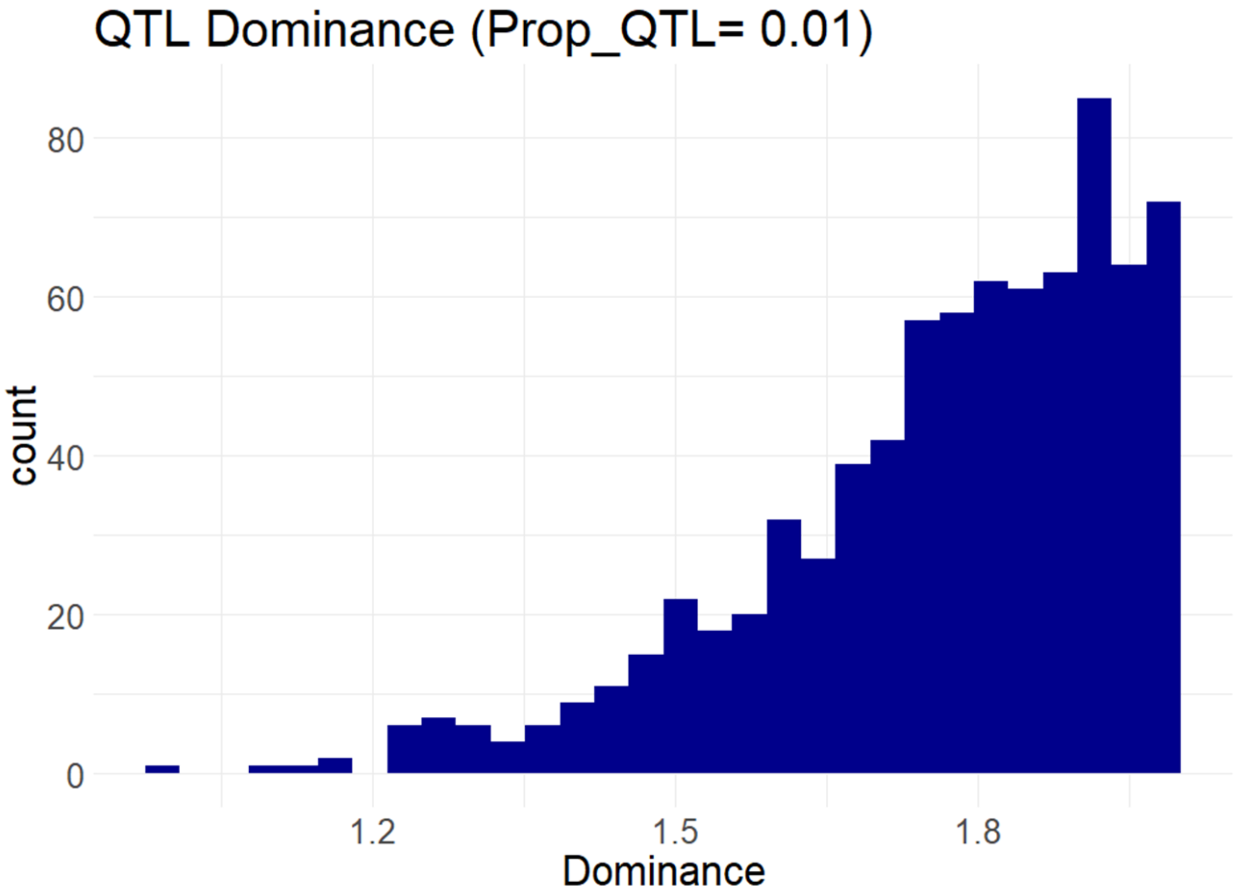

Supplemental Figure 1 QTL Dominance. Distribution of QTL dominance from one simulation instance. A dominance value of 2 indicates a QTL as completely dominant where the value of heterozygous genotype is equal to a homozygous genotype, while a value of 1 indicates a QTL is completely additive, where the value of a heterozygous genotype is half the value of a homozygous genotype.

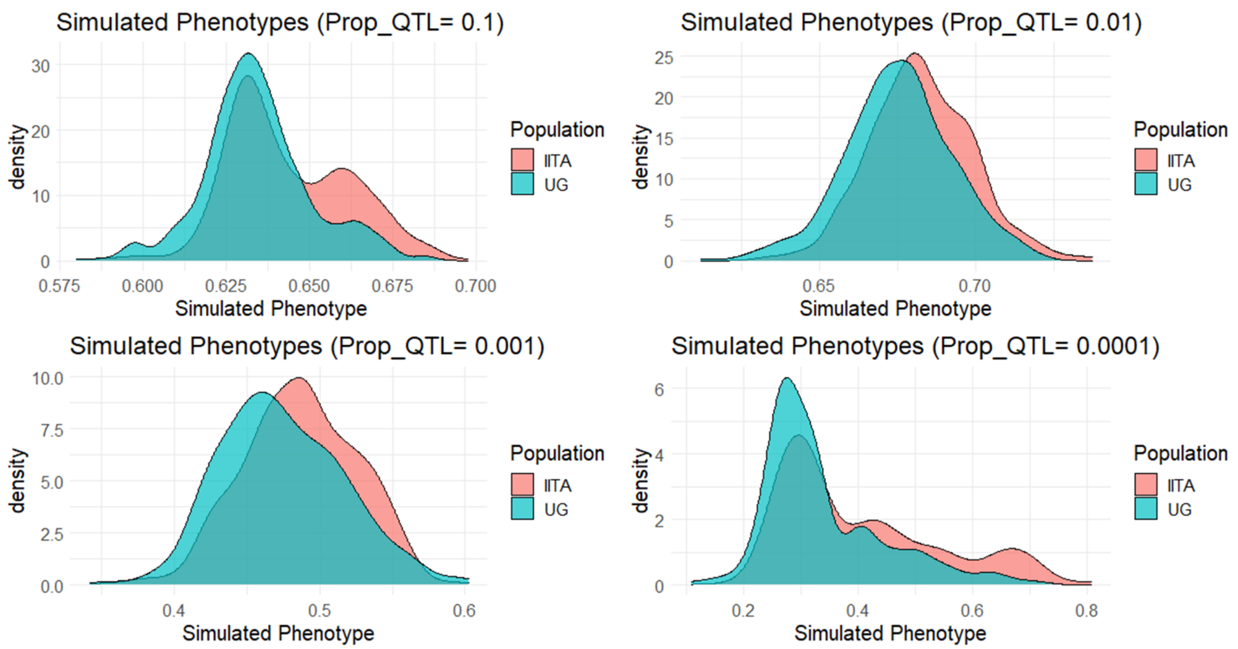

Supplemental Figure 2 Simulated Phenotypes. Phenotypes simulated under one example simulation instance for each genetic architecture.

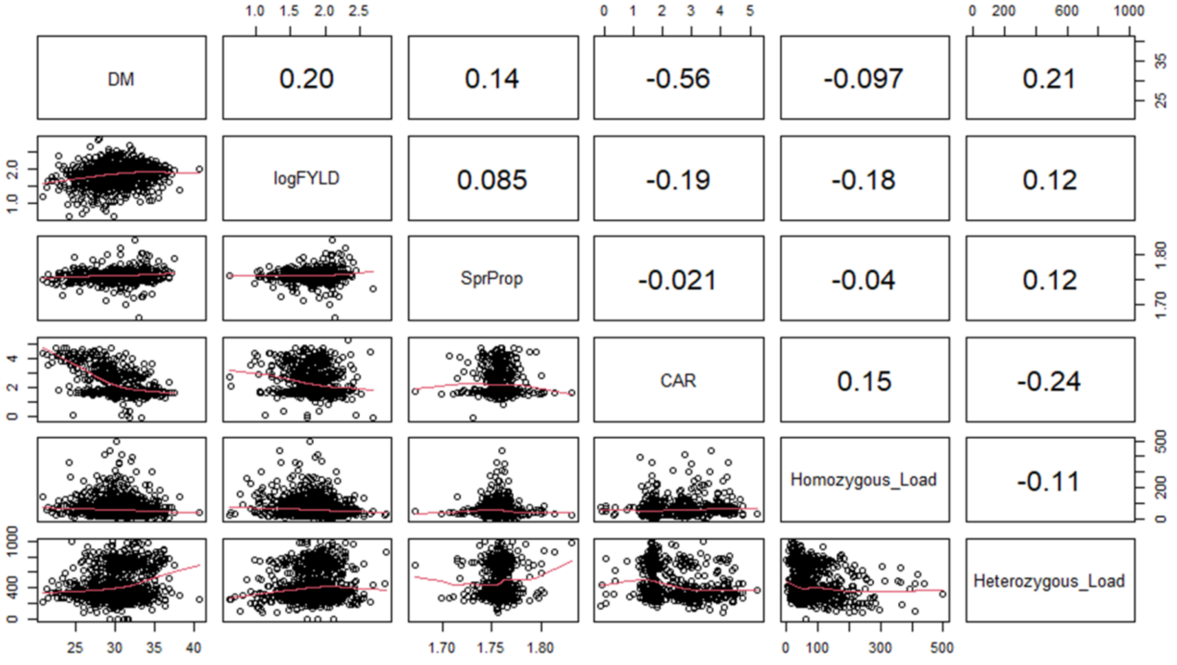

Supplemental Figure 3. Trait and Deleterious Mutation Correlations. Correlations between traits: dry matter percentage (DM), log transformed fresh root yield (logFYLD), proportion of viable sprouts (SprProp), and carotenoid content (CAR). Correlation to homozygous and heterozygous genetic load measured by number of derived deleterious alleles.

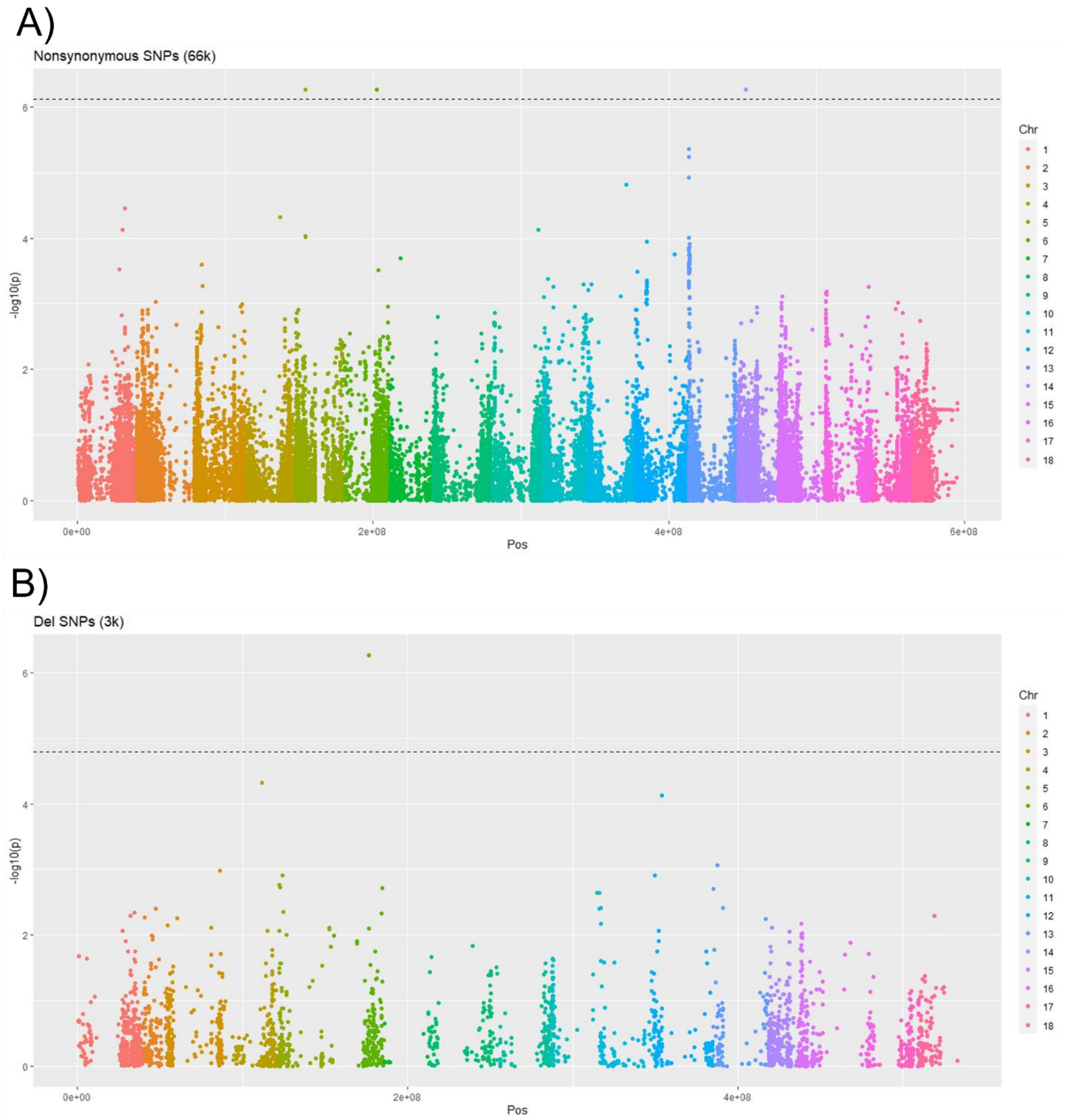

Supplemental Figure 4. Genome Wide Association Fresh Root Yield. Genome wide association using Tassel5 MLM model y~G+PCs(1-5). A) Association performed on 66k Nonsynonymous SNPs, where 5 variants at 3 loci passed Bonferroni significance. B) Association performed on ~3k deleterious SNPs, where 1 variant passing Bonferroni significance.

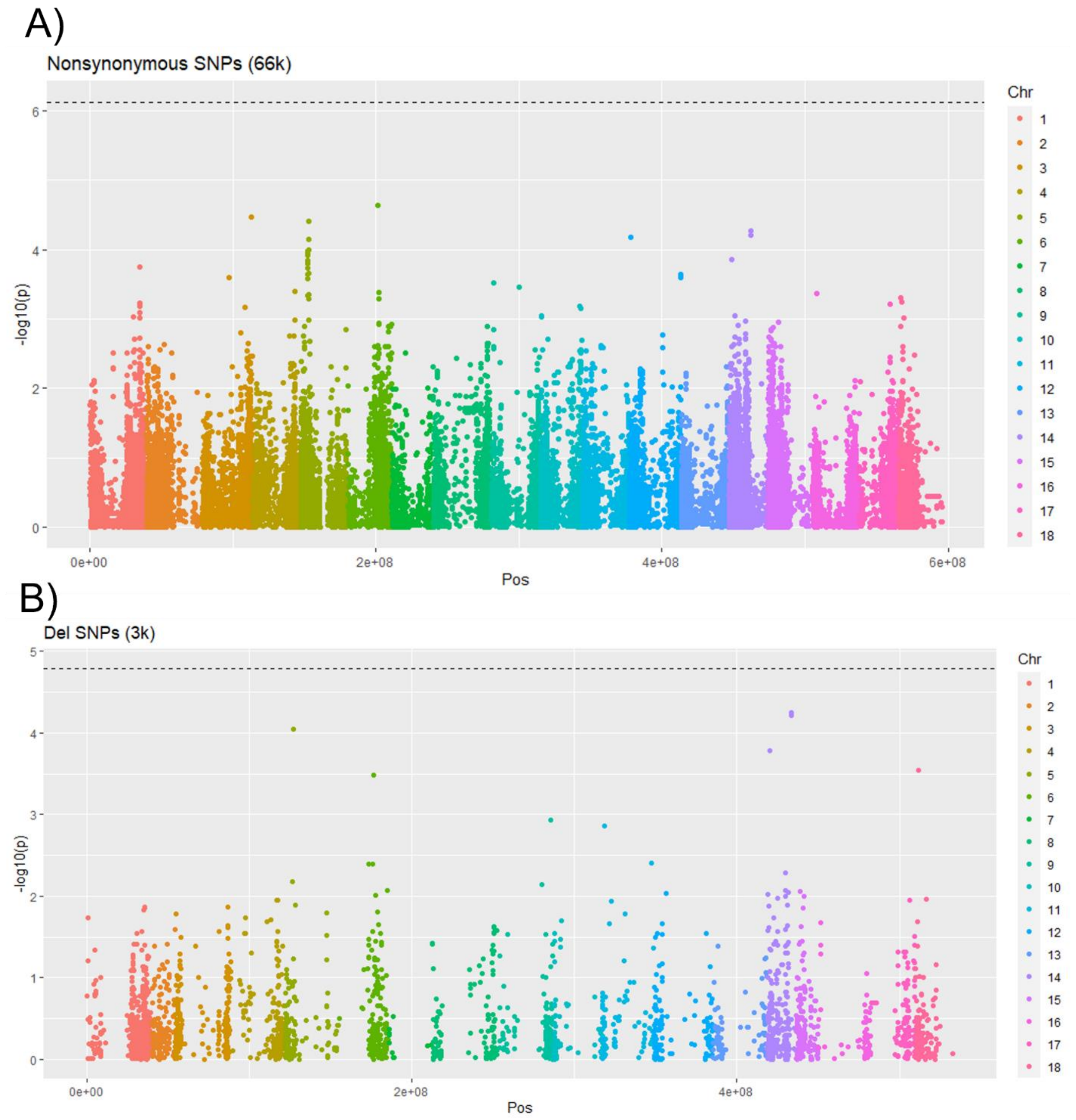

Supplemental Figure 5. Genome Wide Association Dry Matter Percentage. Genome wide association using Tassel5 MLM model y~G+PCs(1-5). A) Association performed on 66k Nonsynonymous SNPs. B) Association performed on ~3k deleterious SNPs.

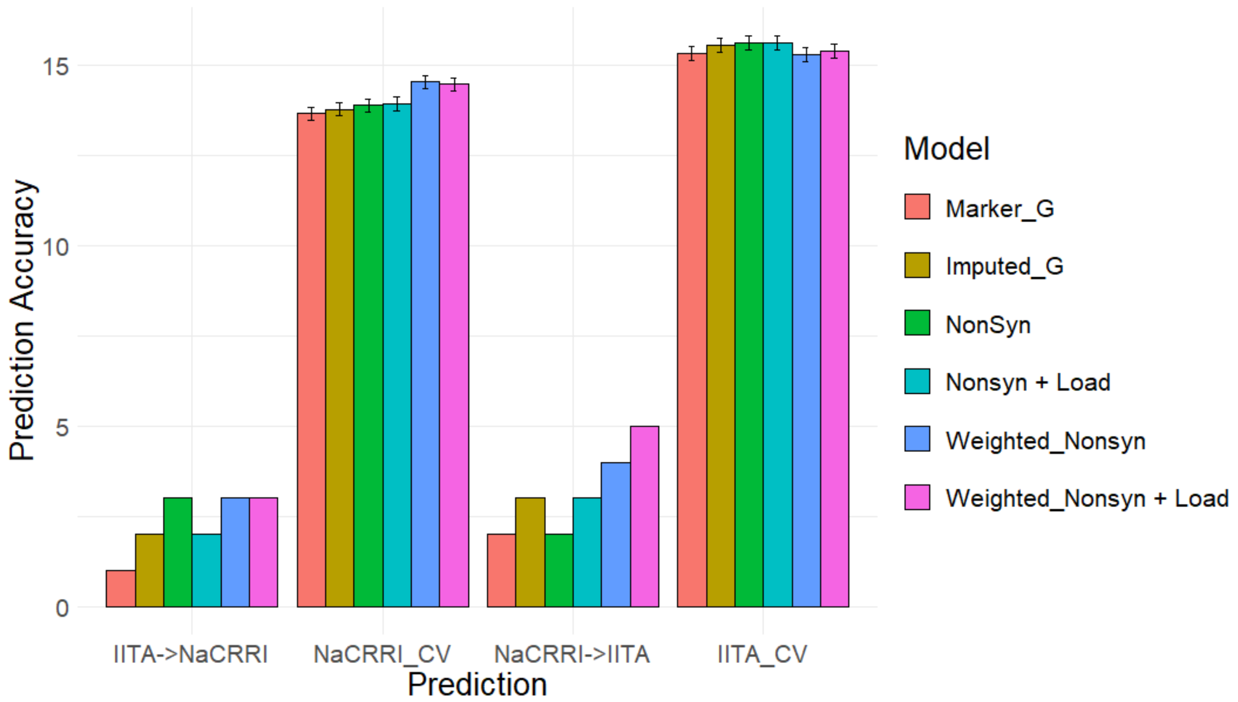

Supplemental Figure 6. Top 25 Prediction Accuracy for Fresh Root Yield. Prediction accuracy is measured by number of 25 predicted clones that are among the top 25 performing clones. Genomic models are represented as bar graph colors where various genomic and deleterious data are used in the genomic prediction. Error bars represent a 95% confidence interval for within-population 10-fold prediction.

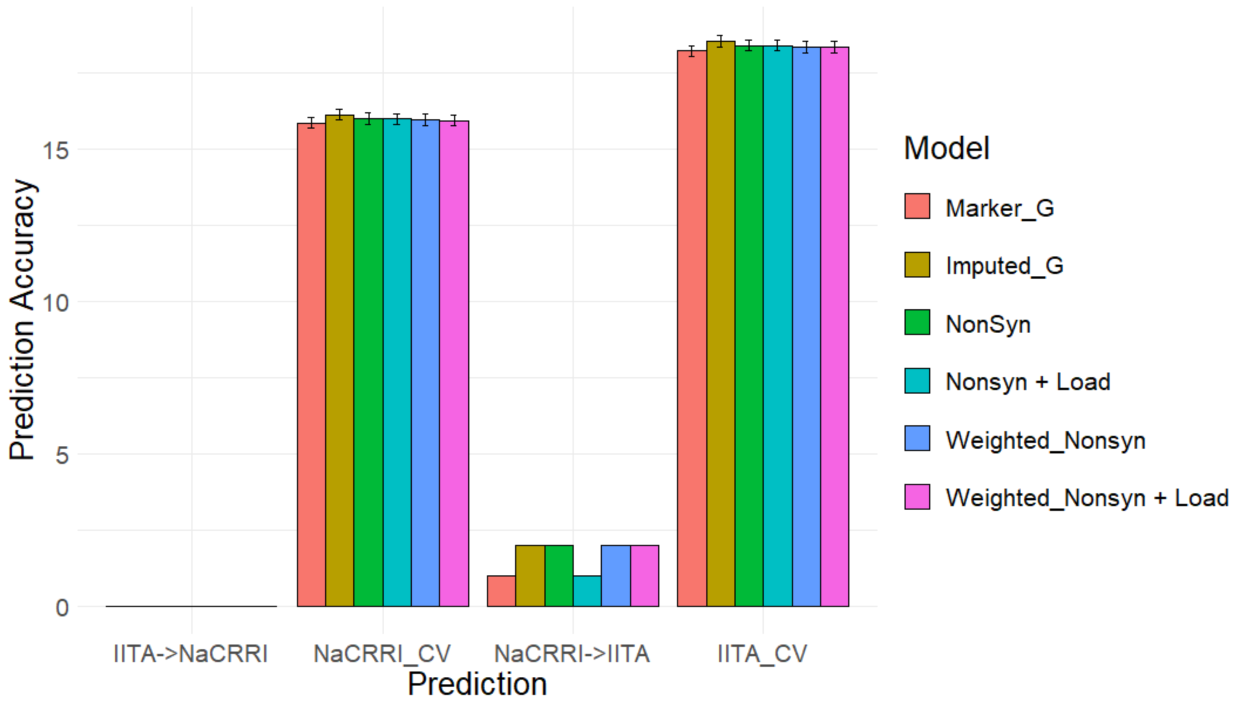

Supplemental Figure 7. Top 25 Prediction Accuracy for Dry Matter percentage. Prediction accuracy is measured by number of 25 predicted clones that are among the top 25 performing clones. Genomic models are represented as bar graph colors where various genomic and deleterious data are used in the genomic prediction. Error bars represent a 95% confidence interval for within-population 10-fold prediction.
